# PCBs and Impaired Cochlear Function in Children: Comparing Pre- and Postnatal Exposures

**DOI:** 10.1289/ehp.122-A310

**Published:** 2014-11-01

**Authors:** Julia R. Barrett

**Affiliations:** Julia R. Barrett, MS, ELS, a Madison, WI–based science writer and editor, has written for *EHP* since 1996. She is a member of the National Association of Science Writers and the Board of Editors in the Life Sciences.

High stability, the quality that made polychlorinated biphenyls (PCBs) so useful in hundreds of industrial and commercial applications during the mid-1900s, has ensured the compounds’ continuing presence in the environment despite decades of banned and restricted use.^1^ This class of more than 200 structurally related chemicals has been linked to both cancer and noncancer outcomes,^1^ and some studies suggest prenatal or early-life exposure to PCBs may adversely affect the auditory system.^2^^,^^3^ A new study reported in *EHP* specifically links postnatal—but not prenatal—PCB exposure with impaired cochlear function.^2^

**Figure:**
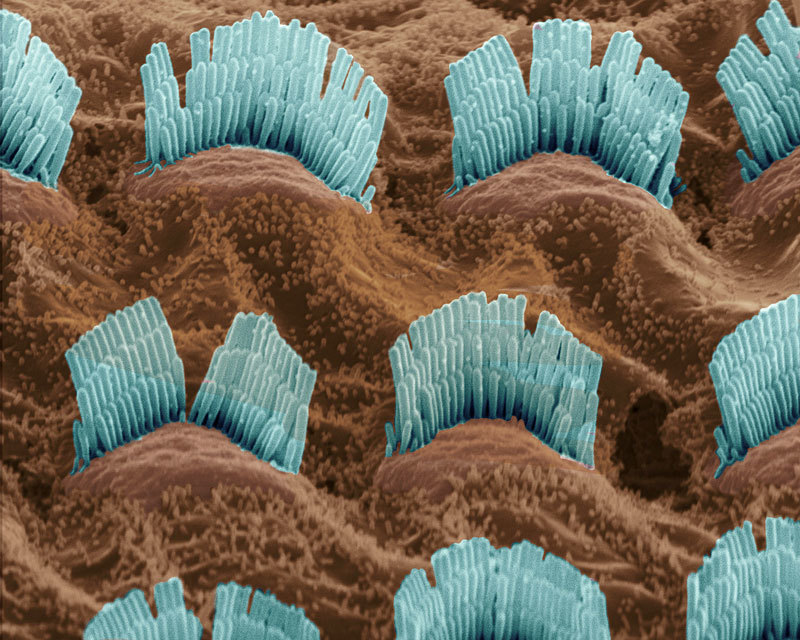
Clusters of hair cells populate the cochlea and detect sounds transmitted to the inner ear. © David Spears FRPS FRMS/Corbis

The cochlea, an extremely sensitive organ in the inner ear, converts soundwave vibrations to nerve impulses transmitted to the brain. Cochlear damage can impair hearing and is a well-known side effect of certain antibiotics and chemotherapeutic drugs.^4^ Rodent studies have shown that PCBs, too, can affect the cochlea, leading to hearing loss.^5^ Some human studies have reported associations between PCB exposure and hearing impairment, particularly in children,^6^^,^^7^ although others have found no such association.^8^

The current study focused on mother–infant pairs from an ongoing birth cohort study in eastern Slovakia. Study participants enrolled during hospital stays for childbirth in 2002–2004. Maternal and cord blood samples were collected for PCB and lipid measurements, and children underwent blood tests at 6, 16, and 45 months of age. At 45 months, the children also underwent otological and auditory testing, including an assessment of distortion product otoacoustic emissions (DPOAEs), a measure of cochlear function.

Health records provided information on the children’s births and subsequent medical visits. Mothers completed questionnaires at study enrollment and at the 16- and 45-month followup visits, providing sociodemographic information and data on lifestyle, diet, medical history, and other factors that could affect their children’s health and hearing. A total of 351 children were included in the study.

Blood tests measured 15 PCB congeners, but analyses focused on PCB-153, which was detected in nearly all samples and closely correlated with total PCB concentrations. An inverse relationship existed between decreased DPOAE amplitude (a marker of diminished cochlear function) and postnatal serum PCB concentrations, with the strongest association observed for cumulative levels of PCBs. The authors suggest this could indicate that duration of exposure, rather than timing, is most important for cochlear development. No association was observed between prenatal PCB exposure and decreased DPOAE amplitude.^2^

The PCB-associated decrease in DPOAE amplitude was not large.^2^ “Certainly, the hearing impairment would be subclinical,” says study coauthor Tomáš Trnovec, a professor of environmental medicine at Slovak Medical University in Bratislava. However, the cochlea is vulnerable to any number of environmental insults, including noise, organochlorine pesticides, and drugs, among others, Trnovec says. “The combined effect of these factors is completely unknown,” he says, adding that “under different settings and other data treatment … a prenatal exposure effect cannot be excluded.”

It’s also unknown whether effects persist and whether continued exposure (combined with other stressors) builds on early damage. “In my opinion, PCB ototoxicity may be involved in the pathogenesis of hearing impairment in the adult population,” says Kyoung-Bok Min, an assistant professor in occupational and environmental medicine at South Korea’s Ajou University School of Medicine, who was not involved in the study. Min and colleagues recently reported an association between PCB serum levels and increased prevalence of hearing impairment in adults who participated in the National Health and Nutrition Examination Survey.^9^ “Further epidemiologic studies are needed in terms of whether the impact of exposure to PCBs on cochlear function changes throughout the life span of the human,” he says.

In addition, Susan Schantz, a professor of veterinary biosciences and psychology at the University of Illinois at Urbana–Champaign, raises the possibility of potential impacts beyond hearing. “One of the things that really interests me—and that I don’t know the answer to—is what does that kind of a decrease [in DPOAE amplitude] mean? Would it be enough to have subtle impacts on language acquisition in children?” asks Schantz, who was not involved in the study. Previous PCB cohort studies have shown an adverse impact on cognitive function, including verbal abilities.^10^ “I’ve always wondered whether subtle changes in hearing could be affecting language development, and then that could be affecting verbal abilities,” Schantz says.

The new study features a particularly strong design based on multiple PCB measurements in a well-characterized population. However, it did not account for potential effects of co-contaminants, which may produce damage when combined with other exposures.^11^ “I think it’s very interesting that they’re seeing reductions to amplitudes related to postnatal exposure,” says Schantz. “That’s not what I would have predicted based on the animal research, but I think it’s very interesting and potentially important.”
